# Trends in firearm injury in a southern California health care system from 2010 to 2020

**DOI:** 10.1186/s12889-023-17116-2

**Published:** 2023-11-10

**Authors:** Margo Sidell, Sonya Negriff, Corinna Koebnick, Deborah Ling Grant, Claudia Nau, Hui Zhou, Rulin Hechter

**Affiliations:** 1 Kaiser Permanente Southern California, 100 S. Los Robles, 2nd Floor, Pasadena, CA 91101 USA; 2 Kaiser Permanente Bernard J. Tyson School of Medicine, Pasadena, CA USA

**Keywords:** Firearm injury, Health care system

## Abstract

**Background:**

Firearm injury is a significant public health concern in the United States.

**Methods:**

Data on fatal and nonfatal firearm injuries were obtained from a cohort of *N* = 7,473,650 members of Kaiser Permanente Southern California, a large integrated healthcare system between 2010 and 2020. Age-adjusted rates of combined fatal and nonfatal firearm injury per 100,000 members were calculated by year, with the 2010 US census as the reference population. Trends were evaluated using Poisson or negative binomial regression.

**Results:**

There was an increasing trend in overall firearm injuries between 2010 and 2020 among adults in this large integrated healthcare system (*p* < .0001), primarily driven by non-self-inflicted firearm injuries (*p* < .0001). Self-inflicted injuries decreased during this time (*p* = .01). Injuries among youth showed no significant change.

**Conclusion:**

There was an increasing trend in firearm injuries between 2010 and 2020 among adults in this large integrated healthcare system, primarily driven by non-self-inflicted firearm injuries; however, self-inflicted injuries decreased during this time. Injuries among youth showed no significant change.

**Supplementary Information:**

The online version contains supplementary material available at 10.1186/s12889-023-17116-2.

## Background

Firearm injury is a significant public health concern in the United States (US) [1–3]. The Centers for Disease Control and Prevention (CDC) track fatal injury data and show around 10.3 firearm-related deaths per 100,000 person-years from 1999 to 2014, with an increase to 11.8 per 100,000 person years 2016–2018 [4]. Additionally, these data show differences in trends based on intent. Specifically, self-inflicted firearm deaths began to increase in 2006 whereas non-self-inflicted firearm deaths including homicides and unintentional injuries increased beginning in 2014 (from 4.3 to 4.7 per 100,000) [5]. Data on nonfatal firearm injuries are more difficult to ascertain as the CDC database only includes a small fraction of the data from emergency departments across the US [2]. To address this issue, a report combining data from the CDC and the Nationwide Emergency Department Sample (NEDS) showed that there was a significant increase in the rate of nonfatal self-inflicted firearm injuries between 2009 and 2017, increasing from 6.1 to 7.3 per 100,000 per year [2]. There were no statistically significant trends in combined rates of fatal and nonfatal firearm injuries between 2009 and 2017, whereas self-inflicted firearm injuries increased during this time [2, 6].

Trends also differ for adults versus youth. Firearm-related injury was the second leading cause of death among youth and adolescents in the US until 2016 and leading cause in 2020 [7, 8]. According to the Nationwide Readmission Database, rates of hospitalization for firearm injury varied between 8.19 and 11.41 per 100,000 between 2010 and 2017, with no apparent increase in trend across time [9]. However, when separating self-inflicted from non-self-inflicted injuries, data from the National Trauma Data Bank (NTDB) showed the trend from 2010 to 2016 decreased for assault from 22.7% to 17.6% but increased from 8.7% to 10.2% for self-inflicted injuries. In addition, the majority of pediatric firearm injuries were due to assaults (52.1%) while 31.5% were determined to be self-inflicted [10]. Updated data are necessary to continue surveillance of trends as well as differentiate changes in the rates for self-inflicted versus non-self-inflicted firearm injury in order to inform policy and public health prevention efforts.

In this study we add to the existing evidence by examining the secular trends in fatal and nonfatal firearm injuries among youth and adults in an integrated health system in Southern California from 2010 to 2020 and compared the trends stratified by injury intention (self-inflicted and non-self-inflicted).

## Methods

### Cohort

Trends in firearm injury from 2010 to 2020 were assessed in a retrospective cohort of Kaiser Permanente Southern California (KPSC) members using electronic medical record (EMR) data. KPSC is a diverse integrated healthcare system whose members approximately represent the socio-characteristics of the Southern California population [11]. Individuals of any age with at least one healthcare encounter between January 1, 2010 and December 31, 2020 were included. Demographics were obtained from administrative data. Member neighborhood education and poverty was proxied using census tract level estimates from the American Community Survey (ACS) 5-year estimates for year of cohort entry. Medicaid status was used as a proxy of low-income as these data were available at the individual level.

### Outcome

Firearm injuries were ascertained by inpatient and outpatient International Classification of Diseases 9^th^ and 10^th^ revision (ICD) diagnosis codes or immediate cause of death from California State Death Master Files (Supplement Table 1).

### Statistical analysis

Age-adjusted rates of combined fatal and nonfatal firearm injury per 100,000 members were calculated by year, with the 2010 US census as the reference population. Regression models with a Poisson distribution or a negative binomial distribution in cases of overdispersion in the data, the natural log of person-days as offset, and year as a covariate were used to assess trend over time using SAS proc genmod. Members were divided into two age strata, youth ages 0–17 and adults 18 + years old based on age at start of each calendar year. Youth turning 18 transitioned to the adult cohort. Injury intention was defined as self-inflicted or non-self-inflicted (unintentional, assault, or undetermined) based on ICD codes. All analyses were performed with alpha = 0.05 two-sided tests using SAS version 9.4 (SAS Institute, Inc., Cary, NC, USA). The study was approved by the KPSC Institutional Review Board and included a waiver of signed consent. All methods were carried out in accordance with relevant guidelines and regulations.

## Results

The cohort (*n* = 7,473,650) was racially/ethnically diverse (10% Asian, 40% Hispanic, 0.2% Native American/Native Alaskan, 7.6% non-Hispanic Black, 32.7% non-Hispanic White, and 0.7% Pacific Islander) with 51.5% women and 72% age 18 years old or higher at cohort entry (Table 1). Of this cohort, 14.3% received Medicaid or Medicare assistance, and 28.6% lived in a neighborhood where < 75% of residents had a high school degree or higher (Table 1). The median follow-up time was 6.1 (IQR 2.5, 11.0) years.

**Table 1:** Characteristics of cohort members enrolled between 2010–2020 in Kaiser Permanente Southern California, No. (%)

	**Total (*N* = **7,473,650**)**
**Age, years**	
0–17	2086939 (27.9)
18 +	5386711 (72.1)
**Gender**	
Female	3849388 (51.5)
Male	3624262 (48.5)
**Race/Ethnicity**	
Asian	747136 (10)
Hispanic	2991678 (40)
Multiple	37474 (0.5)
Native American/ Alaskan	16552 (0.2)
Non-Hispanic Black	571549 (7.6)
Non-Hispanic White	2443922 (32.7)
Pacific Islander	52378 (0.7)
Unknown	612961 (8.2)
**Year of Cohort Entry**	
2010	3453701 (46.2)
2011	457545 (6.1)
2012	402153 (5.4)
2013	365775 (4.9)
2014	429905 (5.8)
2015	466429 (6.2)
2016	408533 (5.5)
2017	416812 (5.6)
2018	404746 (5.4)
2019	357491 (4.8)
2020	310560 (4.2)
**Medicaid or Medicare**	1067042 (14.3)
**Neighborhood Education, Percent with high school degree or higher**	
Missing	619,184 (8.3)
< 50	304,193 (4.1)
50–74.9	1,831,805 (24.5)
75 +	4,718,468 (63.1)
Neighborhood Income, Percent less than $30,000	
Missing	621,977 (8.3)
< 25	4,696,866 (62.8)
25–49.9	2,013,087 (26.9)
50 +	141,720 (1.9)

**Fig. 1:**
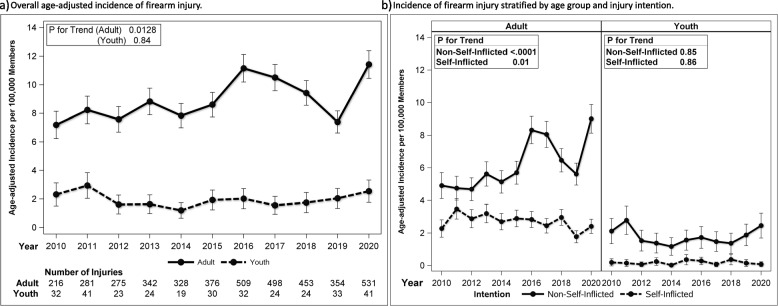
Age-adjusted^a^ incidence per 100,000 members, 95% confidence intervals, and number of combined fatal and nonfatal firearm injuries^b^ by age group per year from 2010 to 2020 **a** overall and **b** stratified by injury intention. ^a^Age-adjusted reference population 2010 US census. ^b^Number of injuries by intention not listed due to small sample size in some years considered protected health information

The incidence of overall firearm injuries increased in adults during the study period, reaching 11.42 injuries per 100,000 members in 2020 (*p* for trend 0.0128) (Fig. 1). The rate per 100,000 members of adult non-self-inflicted injuries increased during the study period from 4.9 (95% CI 4.1, 5.7) in 2010 to 9.0 (95% CI 8.1, 9.9) in 2020 (*p* for trend < 0.0001) with the most profound change from 2019 to 2020 with an increase of 3.4 injuries per 100,000 members. In contrast, the rate of self-inflicted firearm injuries per 100,000 individuals decreased over time (*p* for trend = 0.01) from 2.9 (95% CI 1.7, 2.8) in 2010 to 1.8 (95% CI 1.4, 2.1) in 2019, with a slight increase in 2020 to 2.4 (95% CI 2.0, 2.8) (Fig. 1). The incidence of overall and intent-specific firearm injuries in youth fluctuated over time, but no significant trends were identified during the study period.

## Discussion

Overall, this study showed an increasing trend in firearm injuries between 2010 and 2020 among adults in this large integrated healthcare system, primarily driven by non-self-inflicted firearm injuries. The largest increase was in the COVID-19 pandemic year 2020. In contrast, self-inflicted injuries decreased during this time. Firearm injuries among youth showed no significant change across the study period.

Among adults, the increase in non-self-inflicted firearm injuries is consistent with data from the CDC showing an increase in non-self-inflicted firearm death between 2014–2018 [4]. Our data adds to the existing evidence by providing more recent data to demonstrate continued increases in this type of firearm injury. It should be noted that our data combined fatal and on-fatal firearm injury whereas the CDC data only indicate firearm mortality. Our findings differ from those combining fatal and nonfatal injuries, which showed no significant increase across 2009–2017 for non-self-inflicted injuries [2]. This may be due in part to regional differences when comparing a southern California population to the overall population of the US. In addition, the time periods of these studies were slightly different, with our data extending 4 years further. We noted an increase in firearm injuries in 2020, which may have contributed to our significant trend effects.

Our findings indicate that self-inflicted firearm injuries for adults decreased across 2010–2020, which differs from other data. Data from the CDC show that firearm mortality increased beginning in 2006 [5]. However, our trends are similar to those of US nonfatal self-inflicted injuries, which showed a significant increase from 2009–2017 [2]. Given the majority of our data are comprised of nonfatal injuries this is more comparable than fatal injuries trends.

Lastly, we did not observe any significant trends for youth data. While other data from the NTDB showed decreased firearm injuries for assault and increased self-inflicted injuries from 2010 to 2016, this was observed for nonfatal but not fatal injuries [10]. Given our sample size for youth was smaller, this may have contributed to differences in observed trends. US pediatric firearm deaths have also been shown to increase in 2021 [3]. Because our data did not include complete year data for 2021, we were unable to accurately assess this trend.

We note a number of important limitations of this study, including the observational design and inclusion of only those with health care encounters. These data are also not generalizable outside of the geographic area of this healthcare system. There was the potential for misclassification of intention due to the ICD codes denoting unknown intent; however, the number of injuries coded as such was small and this would not meaningfully change our results. Additionally, although coding changed from ICD-9 to ICD-10 in October 2015, we found minimal impact of the transition in our data. The strengths of this study include an extended time period with rigorous capture of data on firearm injury from electronic health records, including health utilization in emergency departments and trauma care received outside of KPSC through claims. Our analyses are also strengthened by the ability to examine both youth and adults in the same time period using the same data source.

## Conclusion

There was an increasing trend in firearm injuries between 2010 and 2020 among adults in this large integrated healthcare system, primarily driven by non-self-inflicted firearm injuries; however, self-inflicted injuries decreased during this time. Injuries among youth showed no significant change. Overall, these findings bolster the public health significance of firearm injury and highlight the need for continued surveillance to track these trends.

### Supplementary Information

**Additional file 1: Supplement Table 1.** ICD9 and ICD10 diagnosis codes and injury intention included in firearm injury definition.

## Data Availability

The datasets generated and analyzed for the current study are not publicly available due to patient confidentiality but may be made available from the corresponding author on reasonable request.

## Abbreviation

US: United States
KPSC: Kaiser Permanente Southern California
EMR: Electronic medical record
ICD: International Classification of Diseases
ACS: American Community Survey

## References

[1] Goldstick JE, Zeoli A, Mair C, Cunningham RM (2019). US firearm-related mortality: national, state, and population trends, 1999–2017. Health Aff (Millwood).

[2] Kaufman EJ, Wiebe DJ, Xiong RA, Morrison CN, Seamon MJ, Delgado MK (2021). Epidemiologic trends in fatal and nonfatal firearm injuries in the US, 2009–2017. JAMA Intern Med.

[3] Roberts BK, Nofi CP, Cornell E, Kapoor S, Harrison L, Sathya C. Trends and disparities in firearm deaths among children. Pediatrics. 2023;152(3). 10.1542/peds.2023-061296. 10.1542/peds.2023-061296 PMC10471507 37599647

[4] Goldstick JE, Carter PM, Cunningham RM (2021). Current epidemiological trends in firearm mortality in the United States. JAMA Psychiat.

[5] Bailey HM, Zuo Y, Li F (2019). Changes in patterns of mortality rates and years of life lost due to firearms in the United States, 1999 to 2016: a joinpoint analysis. PLoS One.

[6] Centers for Disease Control and Prevention NCfIPaC. Web-based Injury Statistics Query and Reporting System (WISQARS). https://www.cdc.gov/injury/wisqars. Accessed 29 Oct 2021.

[7] Cunningham RM, Walton MA, Carter PM (2018). The major causes of death in children and adolescents in the United States. N Engl J Med.

[8] Goldstick JE, Cunningham RM, Carter PM (2022). Current causes of death in children and adolescents in the United States. N Engl J Med.

[9] Simpson JT (2022). Trends and burden of firearm-related injuries among children and adolescents: a national perspective. J Surg Res.

[10] Cheng T, Burjonrappa S (2022). Pediatric firearm injury trends in the United States: a national trauma data bank (NTDB) analysis. J Pediatr Surg.

[11] Koebnick C, Langer-Gould AM, Gould MK (2012). Sociodemographic characteristics of members of a large, integrated health care system: comparison with US Census Bureau data. Perm J Summer.

