# Supplementary material for: Trends in firearm injury in a southern California health care system from 2010 to 2020
Source: BMC Public Health. 2023 Nov 10;23:2220. doi: 10.1186/s12889-023-17116-2 (PMC10636882; doi:10.1186/s12889-023-17116-2)
Supplement: Supplementary file 1 — Additional file 1: Supplement Table 1. ICD9 and ICD10 diagnosis codes and injury intention included in firearm injury definition. [file 12889_2023_17116_MOESM1_ESM.doc]

**Supplementary Material**

| **Item** | **Page** |
| --- | --- |
| Supplement Table 1. ICD9 and ICD10 diagnosis codes and injury intention included in firearm injury definition. | 2 |
|  |  |

**Supplement Table 1. ICD9 and ICD10 diagnosis codes and injury intention included in firearm injury definition.**

| **ICD Version** | **ICD Code** | **Description** | **Injury Intent** |
| --- | --- | --- | --- |
| ICD9 | E922.0 | ACCIDENT CAUSED BY HANDGUN | Non-Self-Inflicted |
| ICD9 | E922.1 | ACCIDENT CAUSED BY SHOTGUN | Non-Self-Inflicted |
| ICD9 | E922.2 | ACCIDENT CAUSED BY HUNTING RIFLE | Non-Self-Inflicted |
| ICD9 | E922.3 | ACCIDENT CAUSED BY MILITARY FIREARMS | Non-Self-Inflicted |
| ICD9 | E955.0 | CAUSE OF SUICIDE OR SELF INFLICTED INJURY, HANDGUN | Self-Inflicted |
| ICD9 | E955.1 | CAUSE OF SUICIDE OR SELF INFLICTED INJURY, SHOTGUN | Self-Inflicted |
| ICD9 | E955.2 | CAUSE OF SUICIDE OR SELF INFLICTED INJURY, HUNTING RIFLE | Self-Inflicted |
| ICD9 | E955.3 | CAUSE OF SUICIDE OR SELF INFLICTED INJURY, MACHINE GUN | Self-Inflicted |
| ICD9 | E955.4 | CAUSE OF SUICIDE OR SELF INFLICTED INJURY, OTHER AND UNSPECIFIED FIREARM | Self-Inflicted |
| ICD9 | E965.0 | ASSAULT BY HANDGUN | Non-Self-Inflicted |
| ICD9 | E965.1 | ASSAULT BY SHOTGUN | Non-Self-Inflicted |
| ICD9 | E965.2 | ASSAULT BY HUNTING RIFLE | Non-Self-Inflicted |
| ICD9 | E965.3 | ASSAULT BY MILITARY FIREARMS | Non-Self-Inflicted |
| ICD9 | E965.4 | ASSAULT BY OTHER AND UNSPECIFIED FIREARM | Non-Self-Inflicted |
| ICD9 | E985.0 | HANDGUN INJURY | Non-Self-Inflicted |
| ICD9 | E985.1 | SHOTGUN INJURY | Non-Self-Inflicted |
| ICD9 | E985.2 | HUNTING RIFLE INJURY | Non-Self-Inflicted |
| ICD9 | E985.3 | MILITARY FIREARMS INJURY | Non-Self-Inflicted |
| ICD10 | W32.0XXA | ACCIDENTAL HANDGUN DISCHARGE, INITIAL ENCOUNTER | Non-Self-Inflicted |
| ICD10 | W32.1XXA | ACCIDENTAL HANDGUN MALFUNCTION, INITIAL ENCOUNTER | Non-Self-Inflicted |
| ICD10 | W33.00XA | ACCIDENTAL DISCHARGE OF UNSP LARGER FIREARM, INIT ENCNTR | Non-Self-Inflicted |
| ICD10 | W33.01XA | ACCIDENTAL DISCHARGE OF SHOTGUN, INITIAL ENCOUNTER | Non-Self-Inflicted |
| ICD10 | W33.02XA | ACCIDENTAL DISCHARGE OF HUNTING RIFLE, INITIAL ENCOUNTER | Non-Self-Inflicted |
| ICD10 | W33.03XA | ACCIDENTAL DISCHARGE OF MACHINE GUN, INITIAL ENCOUNTER | Non-Self-Inflicted |
| ICD10 | W33.09XA | ACCIDENTAL DISCHARGE OF OTHER LARGER FIREARM, INIT ENCNTR | Non-Self-Inflicted |
| ICD10 | W34.00XA | ACCIDENTAL DISCHARGE FROM UNSP FIREARMS OR GUN, INIT ENCNTR | Non-Self-Inflicted |
| ICD10 | W34.09XA | ACCIDENTAL DISCHARGE FROM OTH FIREARMS, INIT ENCNTR | Non-Self-Inflicted |
| ICD10 | X72.XXXA | CAUSE OF SUICIDE OR SELF INFLICTED INJURY, HANDGUN | Self-Inflicted |
| ICD10 | X73.0XXA | CAUSE OF SUICIDE OR SELF INFLICTED INJURY, SHOTGUN | Self-Inflicted |
| ICD10 | X73.1XXA | CAUSE OF SUICIDE OR SELF INFLICTED INJURY, HUNTING RIFLE | Self-Inflicted |
| ICD10 | X73.2XXA | CAUSE OF SUICIDE OR SELF INFLICTED INJURY, MACHINE GUN | Self-Inflicted |
| ICD10 | X73.8XXA | INTENTIONAL SELF-HARM BY OTH LARGER FIREARM DISCHARGE, INIT | Self-Inflicted |
| ICD10 | X73.9XXA | INTENTIONAL SELF-HARM BY UNSP LARGER FIREARM DISCHARGE, INIT | Self-Inflicted |
| ICD10 | X74.8XXA | INTENTIONAL SELF-HARM BY OTH FIREARM DISCHARGE, INIT ENCNTR | Self-Inflicted |
| ICD10 | X74.9XXA | CAUSE OF SUICIDE OR SELF INFLICTED INJURY, GUNSHOT | Self-Inflicted |
| ICD10 | X93.XXXA | ASSAULT BY HANDGUN DISCHARGE, INITIAL ENCOUNTER | Non-Self-Inflicted |
| ICD10 | X93.XXXA | CAUSE OF ASSAULT, HANDGUN | Non-Self-Inflicted |
| ICD10 | X94.0XXA | CAUSE OF ASSAULT, SHOTGUN | Non-Self-Inflicted |
| ICD10 | X94.1XXA | CAUSE OF ASSAULT, HUNTING RIFLE | Non-Self-Inflicted |
| ICD10 | X94.2XXA | CAUSE OF ASSAULT, MACHINE GUN | Non-Self-Inflicted |
| ICD10 | X94.8XXA | ASSAULT BY OTHER LARGER FIREARM DISCHARGE, INITIAL ENCOUNTER | Non-Self-Inflicted |
| ICD10 | X94.9XXA | ASSAULT BY UNSPECIFIED LARGER FIREARM DISCHARGE, INIT ENCNTR | Non-Self-Inflicted |
| ICD10 | X95.8XXA | ASSAULT BY OTHER FIREARM DISCHARGE, INITIAL ENCOUNTER | Non-Self-Inflicted |
| ICD10 | X95.9XXA | CAUSE OF ASSAULT, GUNSHOT | Non-Self-Inflicted |
| ICD10 | Y22.XXXA | HANDGUN DISCHARGE, UNDETERMINED INTENT, INITIAL ENCOUNTER | Non-Self-Inflicted |
| ICD10 | Y23.0XXA | SHOTGUN DISCHARGE, UNDETERMINED INTENT, INITIAL ENCOUNTER | Non-Self-Inflicted |
| ICD10 | Y23.1XXA | HUNTING RIFLE DISCHARGE, UNDETERMINED INTENT, INIT ENCNTR | Non-Self-Inflicted |
| ICD10 | Y23.2XXA | MILITARY FIREARM DISCHARGE, UNDETERMINED INTENT, INIT ENCNTR | Non-Self-Inflicted |
| ICD10 | Y23.3XXA | MACHINE GUN DISCHARGE, UNDETERMINED INTENT, INIT ENCNTR | Non-Self-Inflicted |
| ICD10 | Y23.8XXA | OTH LARGER FIREARM DISCHARGE, UNDETERMINED INTENT, INIT | Non-Self-Inflicted |
| ICD10 | Y23.9XXA | UNSP LARGER FIREARM DISCHARGE, UNDETERMINED INTENT, INIT | Non-Self-Inflicted |
| ICD10 | Y24.8XXA | OTHER FIREARM DISCHARGE, UNDETERMINED INTENT, INIT ENCNTR | Non-Self-Inflicted |
| ICD10 | Y24.9XXA | UNSP FIREARM DISCHARGE, UNDETERMINED INTENT, INIT ENCNTR | Non-Self-Inflicted |
